# Editorial: Metabolism Meets Function: Untangling the Cross-Talk Between Signaling and Metabolism

**DOI:** 10.3389/fonc.2020.607511

**Published:** 2020-10-20

**Authors:** Alessandra Castegna, Daniel W. McVicar, Annalisa Campanella, Erika M. Palmieri, Alessio Menga, Paolo E. Porporato

**Affiliations:** ^1^ Department of Biosciences, Biotechnologies and Biopharmaceutics, University of Bari, Bari, Italy; ^2^ IBIOM-CNR, Institute of Biomembranes, Bioenergetics and Molecular Biotechnologies, National Research Council, Bari, Italy; ^3^ Laboratory of Cancer Immunometabolism, Center for Cancer Research, National Cancer Institute (NCI), Frederick, MD, United States; ^4^ Department of Molecular Biotechnology and Health Science, Molecular Biotechnology Center, University of Torino, Torino, Italy

**Keywords:** metabolism, cancer, Warburg effect, mitochondria, oncometabolite

Tumor metabolism is a long established field in cancer biology, as the seminal findings of Otto Warburg date back to the 1920s. Since then, the discovery that oncogenes, besides promoting the Warburg effect, modulate anabolic pathways, has prompted scientists to re-evaluate the role that tumor metabolism plays in the neoplastic process. Today, metabolic reprogramming of neoplastic cells is considered a hallmark of cancer, with the discovery that flexibility in the acquisition of various cellular characteristics is supported by specific metabolic pathways. Clinical and pharmacological advances, for example the application of FDG-PET in the clinical setting (1) and the development of novel pharmacological strategies based on antimetabolites (2), provide further support and validation of the role of metabolism in cancer. Here, we present a collection of works with the aim of bringing together work from a variety of scientists across the field of tumor metabolism toward an understanding of how different metabolic pathways are activated in neoplastic and surrounding cells, the mechanisms linking altered metabolism to tumorigenesis and the potential for pharmacological applications.

One of the most prominent metabolic adaptations typical of cancer cells is sustained aerobic glycolysis resulting in the consumption of high amounts of glucose even in the presence of oxygen. For some time, it has been known that highly glycolytic cells typically accumulate what was characterized initially as a by-product, lactate. However, many researchers are now identifying novel properties of lactate, including roles in the cancer–cancer and cancer–stromal shuttles, and as a signaling oncometabolite, as nicely reviewed by Baltazar et al. Similarly, we present work by San-Millán et al. further elaborating on the potential effects of lactate overload by describing its role in supporting tumor aggressiveness, regulating transcriptional signatures associated with proliferation and upregulating oncogenes in breast cancer cells. Lactic acid accumulation also results in a drop in extracellular pH, a feature commonly described in cancer and known to promote aggressiveness (3). Not surprisingly, several signaling pathways independently converge to trigger net acid extrusion decreasing extracellular pH. Work by Malinda et al., reported in our collection, details an example of a signaling pathway regulated this way in cancer, the TGF-β signaling.

To date, despite the well-established knowledge that acidosis is a recurring issue in cancer, limited tools are available to map tumor pH *in vivo* and reveal the spatial distribution of acidic areas within the tumor. In an effort to close this knowledge gap, in our collection Consolino et al. present the use of MRI-CEST imaging to map tumor metabolism. In parallel, Cavallari et al. address the promise of exploiting glycolytic metabolism for clinical purposes by describing novel methods to obtain Hyperpolarized [1-^13^C] Pyruvate for metabolic imaging.

In part as a result of their increased glycolytic flux, tumors require high levels of NAD+. Nicotinamide phosphoribosyltransferase (NAMPT), the rate-limiting enzyme in the NAD+ salvage pathway, is upregulated in many cancers and, as such, pharmacological targeting of NAMPT represents an interesting approach to block cancer growth. In our collection, Heske highlights recent findings describing the effects of NAMPT inhibitors on the non-metabolic functions of malignant cells, supporting utilization of co-targeted therapies consisting of NAMPT inhibitors and other drugs to fully exploit the multiple functions of this enzyme.

The impact of NAMPT biology is not limited to the cancer cells themselves. In a companion contribution in our collection, Audrito et al. describe the peculiar role of NAMPT and nicotinate phosphoribosyltransferase (NAPRT) in cells of the immune system during inflammation. These enzymes are released as soluble factors with cytokine/adipokine/DAMP-like actions in inflammatory settings making them possible “two hit” targets in inflammatory cancers. The authors review the available data concerning the interesting and unique dual roles of this family of enzymes in inflammation.

Despite their extraordinary rates of aerobic glycolysis, tumors are not simply glycolytic cells. As Ordway et al. remind us in this collection, tumors display heterogeneous metabolism as an essential component of their physiologic robustness. Indeed, metabolic plasticity is an essential component of tumor resistance to stress, including stress derived from exposure to chemotherapeutics as described by Desbats et al., or radiation as detailed by Gupta et al. herein. Mitochondria play an important role in conferring this metabolic plasticity, as nicely addressed in our collection by Fiorito et al., who describe the role of iron, and in particular of heme, in cancer. Furthermore, we present work by Sanchez-Martin et al. unraveling the importance of mitochondrial metabolism in cancers by addressing the role of TRAP1, a mitochondrial chaperone protein belonging to the HSP90 family, in controlling cancer metabolism, and defining its role as a potential pharmacological target.

A tumor consists not only of transformed cells, but also on non-transformed stromal cells, such as endothelial cells, fibroblasts and macrophages, that can be recruited and hijacked by cancer cells, promoting tumor progression. Because of the important role they play in tumor malignancy, it is crucial to unravel and understand the complexity of the mechanistic relationships between the various cell types within the tumor that constitute the so-called tumor microenvironment (TME) (4). In our collection, Comito et al. describe the metabolic remodeling of the different cell populations within the TME, focusing on reciprocal re-education through the symbiotic sharing of metabolites, acting both as nutrients and transcriptional regulators, and evaluating their impact on tumor growth and metastasis. In addition, Nazemi and Rainero describe how cancer-associated fibroblasts of the TME dictate cancer cell metabolism, describing the impact of nutrient scavenging from the microenvironment in cancer cell growth. Their contribution also focuses on the cross-talk between nutrient signaling and the trafficking of the extracellular matrix (ECM) receptors of the integrin family; critical aspects of tumor aggressiveness.

An important stromal component of the TME are tumor-associated macrophages (TAMs) (5, 6), which contribute to several steps in the formation of metastasis (7, 8), and are recruited through mechanisms that can be mediated by functionally relevant metabolic reprogramming. Identification of the metabolic checkpoints regulating macrophage function, which might be targeted to improve cancer specific immune responses is now a promising strategy for therapeutic intervention. The metabolic mechanisms of macrophage polarization and functional skewing are starting to emerge (9–11), but more needs to be unraveled with respect to trace elements. Metal ions are involved in various biological processes. In our collection, Serra et al. describe new findings regarding the role of these micronutrients in metabolic and cellular signaling mechanisms in macrophages. The processes they describe are components of the “metallic” cross-talk between macrophages and cancer cells and may represent opportunities for innovative pharmaceutical or dietary interventions in cancer therapy.

Amino acid metabolism is also crucial for cancer development. Activation of signaling pathways associated with proliferation can lead to amino acid depletion. Furthermore, amino acid deprivation occurring in cancer tissues might be overcome by the ability of cancer cells to synthesize the given specific amino acid. Herein, Chiu et al. nicely address the role of asparagine synthase (ASNS) and asparagine in cancer. Asparagine biochemistry is gaining more attention in the scientific community with the revelation that ASNS is overexpressed in some cancers, promoting cell proliferation, chemoresistance, and metastasis formation. During proliferation, amino acid metabolism is upregulated *via* the mTOR pathway, a signaling network known to contribute to cancer progression. Different mechanisms and factors regulate mTOR function. In our collection Gozzelino et al. elaborate on the role of one of these, phosphatidylinositol 3-4bisphosphate (PI(3,4)P2), showing it to be a novel emerging signaling molecule that regulates biological functions, and acts as an effector of metabolic reprogramming events relevant in cancer development.

Taken together, the work in the present collection represent a unique contribution to our understanding of the mechanisms, and the functional outcomes, associated with metabolic reprogramming in cancer.

## Author Contributions

AC and PEP drafted the paper. AM, EP, and ALC critically read and edited. AC and DMV finalized the paper. All authors listed have made a substantial, direct, and intellectual contribution to the work, and approved it for publication. 

## Funding

This work was funded, in part, by the intramural research program of the NIH, Center for Cancer Research of the National Cancer Institute (DMV) and from AIRC (MFAG 21564-PEP).

## Conflict of Interest

The authors declare that the research was conducted in the absence of any commercial or financial relationships that could be construed as a potential conflict of interest.
